# Effects of different macromolecular models on reproducibility of FID‐MRSI at 7T

**DOI:** 10.1002/mrm.27922

**Published:** 2019-08-08

**Authors:** Eva Heckova, Michal Považan, Bernhard Strasser, Stanislav Motyka, Gilbert Hangel, Lukas Hingerl, Philipp Moser, Alexandra Lipka, Stephan Gruber, Siegfried Trattnig, Wolfgang Bogner

**Affiliations:** ^1^ High Field MR Centre Department of Biomedical Imaging and Image‐guided Therapy Medical University of Vienna Vienna Austria; ^2^ Russell H. Morgan Department of Radiology and Radiological Science The John Hopkins University School of Medicine Baltimore Maryland; ^3^ Athinoula A. Martinos Center for Biomedical Imaging Department of Radiology Massachusetts General Hospital Harvard Medical School Boston Massachusetts; ^4^ Christian Doppler Laboratory for Clinical Molecular MR Imaging Vienna Austria

**Keywords:** brain, macromolecules, MR spectroscopic imaging, parameterization, reproducibility, ultrahigh field

## Abstract

**Purpose:**

A properly characterized macromolecular (MM) contribution is essential for accurate metabolite quantification in FID‐MRSI. MM information can be included into the fitting model as a single component or parameterized and included over several individual MM resonances, which adds flexibility when pathologic changes are present but is prone to potential overfitting. This study investigates the effects of different MM models on MRSI reproducibility.

**Methods:**

Clinically feasible, high‐resolution FID‐MRSI data were collected in ~5 min at 7 Tesla from 10 healthy volunteers and quantified via LCModel (version 6.3) with 3 basis sets, each with a different approach for how the MM signal was handled: averaged measured whole spectrum (full MM), 9 parameterized components (param MM) with soft constraints to avoid overparameterization, or without any MM information included in the fitting prior knowledge. The test–retest reproducibility of MRSI scans was assessed voxel‐wise using metabolite coefficients of variation and intraclass correlation coefficients and compared between the basis sets. Correlations of concentration estimates were investigated for the param MM fitting model.

**Results:**

The full MM model provided the most reproducible quantification of total NAA, total Cho, myo‐inositol, and glutamate + glutamine ratios to total Cr (coefficients of variations ≤ 8%, intraclass correlation coefficients ≥ 0.76). Using the param MM model resulted in slightly lower reproducibility (up to +3% higher coefficients of variations, up to −0.1 decreased intraclass correlation coefficients). The quantification of the parameterized macromolecules did not affect quantification of the overlapping metabolites.

**Conclusion:**

Clinically feasible FID‐MRSI with an experimentally acquired MM spectrum included in prior knowledge provides highly reproducible quantification for the most common neurometabolites in healthy volunteers. Parameterization of the MM spectrum may be preferred as a compromise between quantification accuracy and reproducibility when the MM content is expected to be pathologically altered.

## 1 INTRODUCTION

MRSI combines the anatomical and biochemical information of an examined tissue and has been extensively used to detect in vivo metabolite levels in various brain disorders.^1^ MRSI benefits especially from ultrahigh field strength (i.e., ≥7 Tesla [T]) as the spectral resolution improves significantly.^2^ However, at higher field strengths the T_2_ relaxation times are shorter, leading to increased T_2_‐related SNR loss for MRSI with long TEs. To maximize the number of detectable signals and overcome some challenges that arise from the ultrahigh field (e.g., chemical shift displacement error), FID‐based MRSI with no echo time but with a negligible acquisition delay was proposed and has received increasing attention in the research community.^3^, ^4^, ^5^, ^6^, ^7^ While the detectability of neurometabolites, such as myo‐inositol (mIns), glutamate (Glu), glutamine (Gln), or glutathione (GSH) improves, FID‐MRSI is also highly sensitive to the broad background macromolecular (MM) signal. The presence of these strong MM resonances poses a challenge for accurate metabolite quantification.^8^, ^9^ To use FID‐MRSI as a reliable tool in a clinical context, it is mandatory to either decrease the MM contribution or include it in the fitting model appropriately while also providing sufficient flexibility for cases in which the MM profile is altered.

One approach is to measure the MM signal separately and subtract it from the metabolite spectrum.^10^, ^11^ Although this is the most direct method with which to identify a subject‐specific MM contribution, it is also motion‐sensitive, prolongs the acquisition time (particularly in case of high‐resolution MRSI), and reduces the SNR, which can eventually decrease the quantification accuracy.

Alternatively, prior knowledge of the MM contribution can be introduced into the fitting process. In case of LCModel, a widely used quantification software, the macromolecules can be approximated by spline functions^12^, ^13^ or experimentally acquired and averaged over a group of healthy volunteers. Studies conducted at 3T have revealed that a measured MM spectrum is superior to a mathematical estimation in terms of MRSI reproducibility because the spline baseline cannot adequately characterize the complicated MM lineshapes.^14^ This is even more pronounced at higher field strengths (≥ 7 T) in which the apparent linewidth of the individual MM resonances approaches that of the J‐coupled metabolites.^15^, ^16^

The most common strategy for measuring the MM spectrum in vivo is to use an inversion recovery sequence, thereby exploiting the short T_1_ relaxation times of macromolecules in contrast to those of most brain metabolites.^8^, ^17^, ^18^, ^19^ Another approach to capture the macromolecules is to back‐extrapolate the metabolite signal and separate it from the MRSI data.^20^ Afterward, the measured MM spectrum either can be directly included in the basis set as a single component or parameterized and included as several individual MM components, which adds flexibility when the MM content and composition change, for example, when pathologic changes are present.^21^, ^22^, ^23^ However, the inclusion of several MM components increases the degrees of freedom of LCModel analysis, which presumably could lead to inaccurate metabolite estimations and negatively influence the reliability of the quantification. This can be avoided by the application of soft constraints. Both approaches, using a whole MM spectrum^17^ or several parameterized MM components,^24^ were evaluated for fairly high SNR (thus, long scan times). Although the precision of metabolite quantification was investigated, the effects on reproducibility were not assessed. Yet, the knowledge about variation in repeated measurements is a critical prerequisite for future application of FID‐MRSI in (longitudinal) clinical studies.

The aim of the present study was therefore to determine the effect of using different MM models on test–retest reproducibility for data acquired via clinically feasible ~5 min FID‐MRSI protocols at 7T.

## 2 METHODS

### 2.1 Subjects

The study was approved by the local ethics board. Ten healthy volunteers (3 females, 7 males; mean age, 28 ± 4 years) were recruited. Informed, written consent was obtained from all subjects prior to the examination. Each subject was measured twice (i.e., test and retest measurement), preferably on the same day. All subjects were removed from the scanner between the sessions. In 4 subjects, the retest measurements were performed on a different day (up to 7 days) due to restricted scanner availability.

### 2.2 Data acquisition

In each subject, test and retest MRI/MRSI scans were performed on a 7T Magnetom MR scanner (Siemens Healthcare, Erlangen, Germany) using a 32‐channel receive array coil combined with a transmit volume coil (Nova Medical, Wilmington, MA). In each session 3D T_1_‐weighted MP2RAGE images were acquired to guide the positioning of MRSI slices and to derive the brain tissue type and structural maps. The spectroscopic data were obtained in 2 single‐slice FID‐MRSI^3^, ^5^ acquisitions in order to test the performance of the sequence over larger brain volume. The first slice was positioned in the transverse plane above the corpus callosum, and the second slice was positioned 12 mm above the first slice. Consistent positioning between test and retest measurement sessions was achieved by using an automatic alignment sequence,^25^ reloading the spectroscopic sequence from the test measurement, and confirmed by visual inspection of the grid position. The parameters of the FID‐MRSI sequence were as follows: acquisition delay/TR of 1.3 ms/600 ms, FOV of 200 × 200 mm^2^, matrix size of 64 × 64, nominal voxel volume of 3.4 × 3.4 × 8 mm^3^, flip angle of 45°, 1024 complex spectral data points, acquisition bandwidth of 6000 Hz, water suppression enhanced through T_1_ effects (WET), 6‐fold 2D‐Controlled Aliasing in Parallel Imaging Results in Higher Acceleration (CAIPIRINHA)^26^ parallel imaging acceleration, and acquisition time of 5:11 min.

### 2.3 Spectroscopic data processing

Brain masks were extracted from T_1_‐weighted images using a brain extraction tool.^27^ MRSI data within the brain masks were processed automatically using a script written in MatLab (version R2013a; MathWorks, Inc., Natick, MA) and Bash (version 4.2.25, Free Software Foundation, Boston, MA). The processing included a multichannel spectroscopic data combined by matching image calibration data (MUSICAL) coil combination of the raw data,^28^ parallel‐imaging reconstruction, Hamming filtering, removal of lipid signal via L_2_‐regularization,^29^ and fitting of the individual spectra with LCModel (version 6.3; LCModel Inc., Oakville, ON, Canada). For this purpose, 3 different basis sets were used, each consisting of 15 metabolite resonances simulated in NMR Scope (jMRUI 5.0), as well as the following:

1. full MM: A single in vivo MM spectrum obtained in previous study^17^ by nulling of brain metabolite signals via double inversion recovery (2 inversion 40 ms Wurst pulses, acquisition delay/TR of 1.3 ms/879 ms, TI_1_ of 570 ms, TI_2_ of 21 ms, flip angle of 55°, matrix size of 32 × 32, nominal voxel volume of 5.6 × 5.6 × 12 mm^3^, 2048 complex spectral data points, acquisition bandwidth of 6000 Hz, water suppression enhanced through T_1_ effects [WET]), removing the residual metabolite signals using advanced method for accurate, robust, and efficient spectral fitting (AMARES),^30^ as described by Craveiro et al.,^31^ and averaging over different brain regions and healthy volunteers.
2. param MM: Nine individual MM components extracted from measured in vivo metabolite‐nulled spectra, with applied soft constraints (via LCModel parameter CHRATO) to avoid overparameterization of the fitting model (detailed description of the parameterization can be found in Považan et al.^24^).
3. no MM: With no macromolecular information included.

Spectral analysis was performed in a frequency range of 0.2 to 4.2 ppm when using the full MM and the param MM basis set. In case of no MM basis set, the frequency range was reduced to 1.8 to 4.2 ppm to avoid the lipid region and MM peaks below. The LCModel parameter DKNTMN, controlling the stiffness of the spline baseline, was set to the default value of 0.15 in all cases. The LCModel control files for the 3 quantification approaches are provided in Supporting Information Text S1.

### 2.4 Data evaluation

The spectral and fitting quality of the data was assessed via 3 parameters: Cramér‐Rao lower bounds (CRLB) of the metabolites reported by LCModel and SNR and FWHM of the fitted NAA peak. Spectra with CRLB_NAA_ > 20% or FWHM_NAA_ > 20 Hz were excluded from further analysis and display (i.e., <2% of all spectra). The excluded spectra were generally located at the periphery of the brain. Metabolite maps were derived from the LCModel quantification results and displayed by MINC software (version 2.0, McConnell Brain Imaging, Montreal, QC, Canada).

For the evaluation, 5 brain regions were defined: frontal white matter (WM), frontal gray matter (GM), parietal WM, parietal GM, and subcortical WM. As a first step, tissue‐type segmentation (GM, WM, and CSF) and structural registration (frontal lobe, parietal lobe, and subcortical WM) were performed on T_1_‐weighted images of both test and retest measurements using automated segmentation tool (FAST) and linear image registration tool (FLIRT) of the FSL package.^32^ To match the point‐spread function of MRSI and T_1_‐weighted MRI, segmented high‐resolution images were Fourier‐transformed to k‐space, matched to the spatial frequency characteristics of the MRSI data, and converted back to image‐space. Only voxels with a minimum of 80% of WM or GM content were used for analysis. The segmented images in MRSI resolution were then combined to create binary masks of the 5 aforementioned regions. Finally, the intersections of binary masks from the test and retest of each subject were used as the final masks.

Statistical analysis and calculations were performed in MatLab (MathWorks) using voxel‐wise analysis. The test–retest reproducibility of metabolite ratio levels was established by an intrasubject coefficient of variation (CV) calculated for each eligible voxel as the SD of the 2 measurements divided by their mean. CVs were grouped to 5 regions according to the predefined binary masks. As a measure of method reliability, the intraclass correlation coefficients (ICC) using an absolute‐agreement, 2‐way, mixed‐effects model were calculated between test and retest. For the param MM basis set, a correlation diagram was derived to investigate whether the concentrations of metabolites and underlying individual MM components were independent of each other. To test for differences in CV, ICC, metabolite ratios, and quantification precision between the 3 basis sets, a nonparametric Friedman test, tailored for comparison of multiple related samples, was used. Subsequently, post hoc analysis by Wilcoxon signed‐ranks tests was performed for pairwise comparisons. The differences between brain regions were compared using Kruskal‐Wallis tests followed by Mann‐Whitney post hoc analysis. Bonferroni correction for multiple testing was applied, and a *P* < 0.05 was considered significant.

## 3 RESULTS

### 3.1 Spectral quality

**Figure 1:**
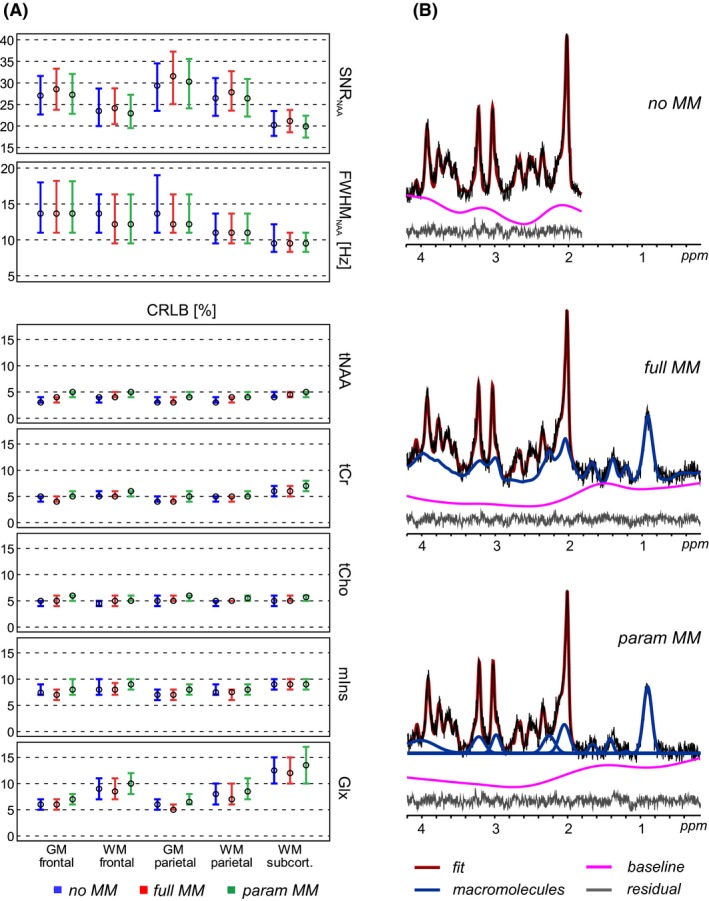
(A) Spectral quality (SNR, linewidth) and quantification precision (Cramér‐Rao lower bounds) parameters. The circles and errror bars show the 25th percentile, median, and the 75th percentile of the total number of analyzed voxels. (B) Representative spectra obtained by FID‐MRSI and quantified in LCModel by 3 different MM prior knowledge models: no MM, with no MM information; full MM, with a single measured MM spectrum; and param MM, with 9 individual MM components included. The fitted baselines have been subtracted from the metabolite and MM spectra. These subtracted baselines and the residua are displayed separately below each LCModel fit. MM, macromolecular

Altogether, 7532 pairs of test–retest spectra collected from 10 subjects were evaluated. Each spectrum was quantified in LCModel by using 3 different basis sets. Of the first MRSI acquisition, 630, 1085, 679, 779, or 1957 spectra were assigned to the frontal GM, frontal WM, parietal GM, parietal WM, or subcortical WM region, respectively. In case of the second MRSI acquisition, 656, 1151, 337, or 258 spectra were assigned to the frontal GM, frontal WM, parietal GM, or parietal WM region, respectively. The subcortical WM of the second MRSI scan was not evaluated because of a small number of assigned spectra. Region‐specific spectral quality parameters are displayed in Figure 1A. The overall spectral quality was high, with the lowest median SNR > 20 in the subcortical WM and the highest median FWHM <14 Hz in the frontal GM. The use of different fitting models had no impact on the metrics.

### 3.2 Quantification precision

Sample spectra fitted by different basis sets are displayed in Figure 1B. The CRLBs were consistently low among the 2 MRSI slice positions and brain regions (except for ~5% higher CRLB_Glx_ in the subcortical WM), as well as between test–retest measurements (median CRLB_tNAA_ ≤ 5%, median CRLB_tCho_ ≤ 6%, median CRLB_tCr_ ≤ 7%, median CRLB_mIns_ ≤ 8%, median CRLB_Glx_ ≤ 14%). The regional differences in CRLB_Glx_ (CRLB_GM_ < CRLB_WM_ < CRLB_subcorticalWM_) were associated with SNR loss (SNR_GM_ > SNR_WM_ > SNR_subcorticalWM_). We observed small alterations in CRLBs among the basis sets (param MM resulted in a +1% increase in CRLBs) (Figure 1A).

**Figure 2:**
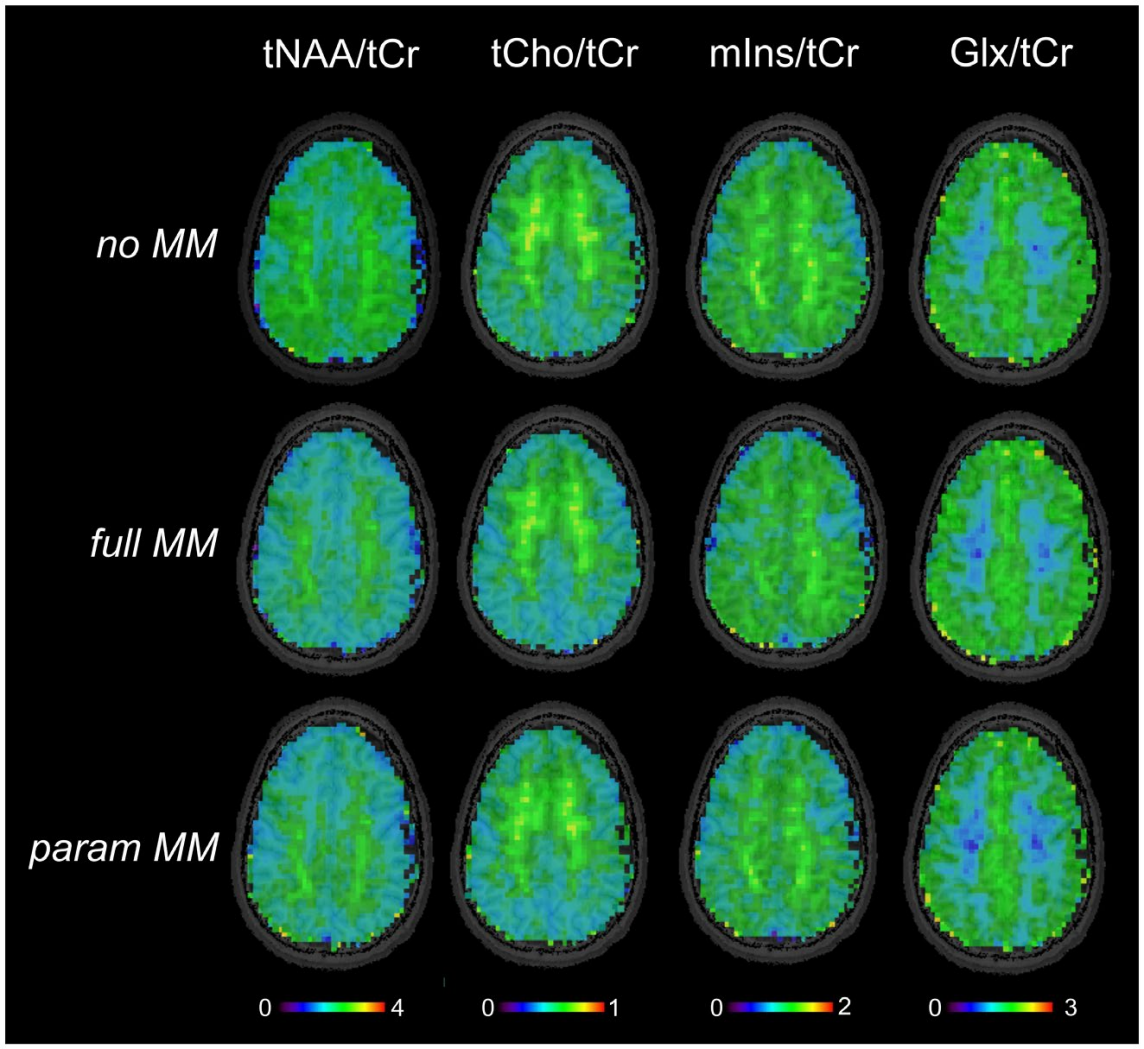
Metabolite maps of one volunteer obtained by FID‐MRSI with 64 × 64 resolution. The quantification of spectra was performed in LCModel using 3 basis sets with different macromolecular prior knowledge included: with no MM information, with a single in vivo MM spectrum (full MM), and with 9 parameterized MM components (param MM) with soft constraints

Sample metabolite maps are displayed in Figure 2. The metabolite ratio levels are summarized in Supporting Information Table S1. The levels of tNAA/tCr in GM (by −13%) and in WM (by −8%), as well as mIns/tCr in WM (by −6%) and Glx/tCr in WM (by −5%), were significantly decreased when using full MM in comparison to no MM scheme (all *P* < 0.001). The differences between full MM and param MM were insignificant, except of ~3% difference in GM of Glx/tCr (*P* = 0.01).

**Figure 3:**
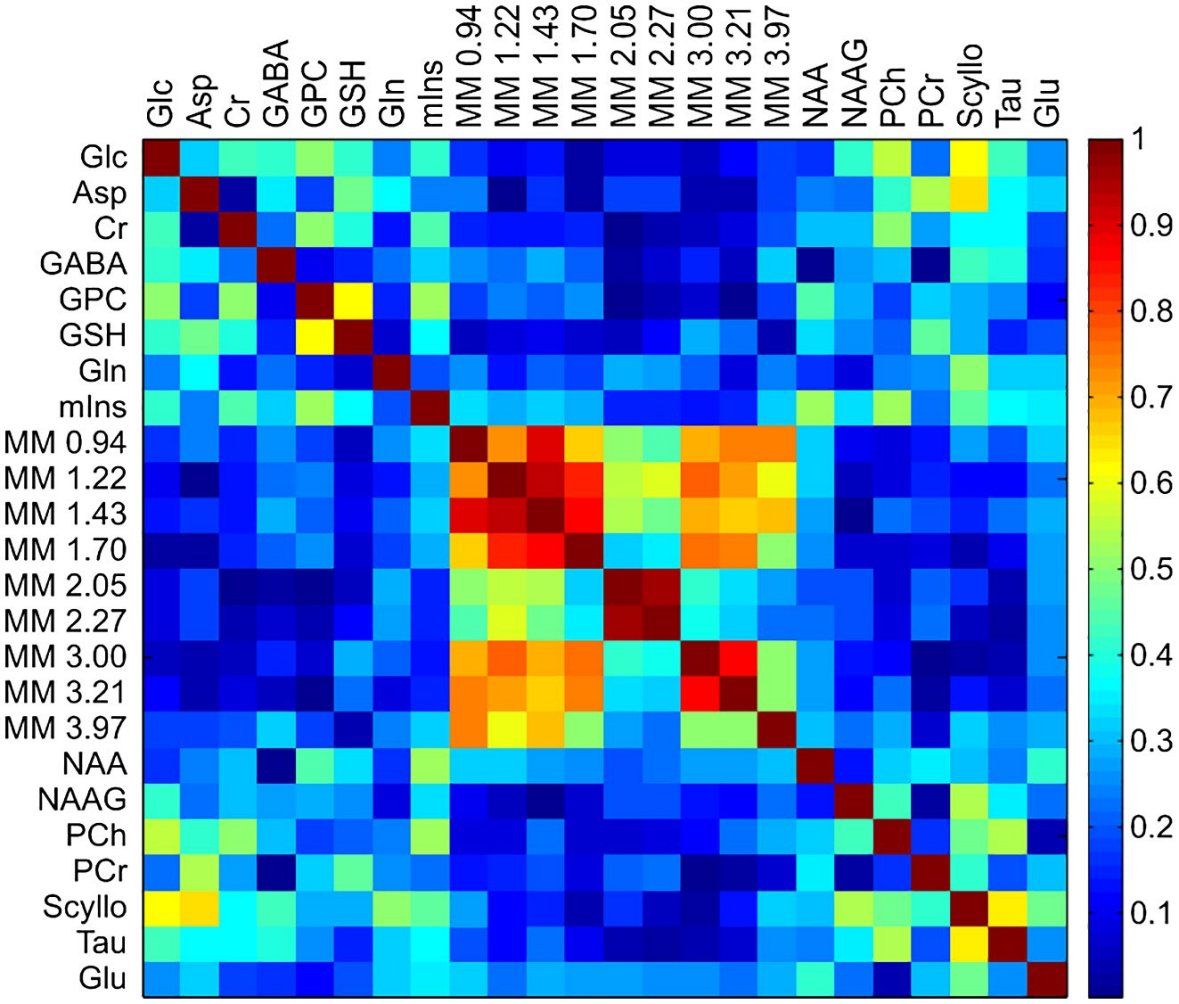
Correlation coefficients derived from quantification results of the LCModel analysis of FID‐MRSI using the basis set that included 9 individual parameterized MM components (param MM). Each element of the matrix indicates the absolute value of the correlation coefficient between the fitting results of the 2 corresponding metabolites

The correlation analysis revealed that the signal amplitudes of several MM resonances were highly interdependent; however, there were no associations or only very weak correlations between individual parameterized macromolecules and overlapping metabolites (Figure 3).

### 3.3 Reproducibility

**Figure 4:**
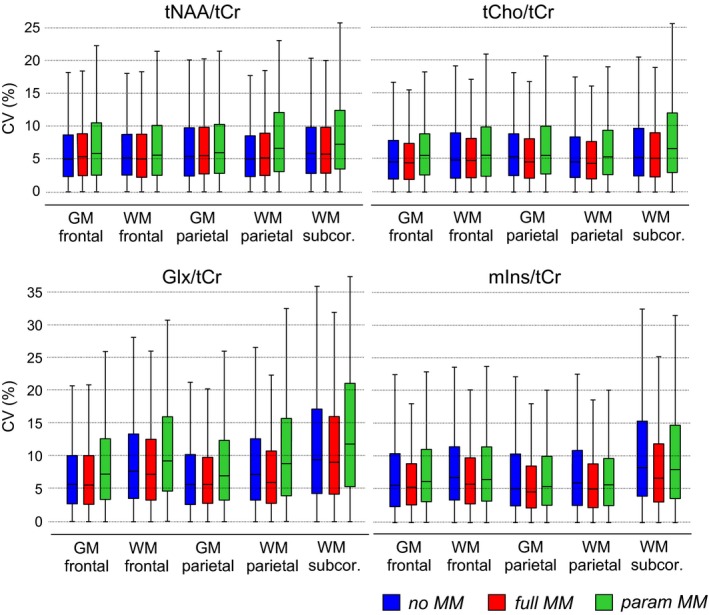
CV values for the voxel‐based analysis of the metabolite ratios obtained by different MM prior knowledge included in the basis sets: no MM, with no MM information; full MM, with a single measured MM spectrum; and param MM, with 9 individual MM components with soft constraints. The box‐and‐whisker‐plots represent the minimum, 25th percentile, median, 75th percentile, and maximum number of the analyzed voxels. CV, coefficient of variations

Figure 4 and Supporting Information Figure S1 summarize the measures of reproducibility for the 4 main metabolite ratios of different fitting approaches. The obtained CVs were relatively consistent and low between the no MM, full MM, and param MM basis sets. The full MM analysis was the most reproducible, with mean CV_tNAA/tCr_ = 7.0%, mean CV_tCho/tCr_ = 5.9%, mean CV_mIns/tCr_ = 7.0%, and mean CV_Glx/tCr_ = 8.1%. Using the param MM scheme yielded up to a +3% increase in CVs compared to full MM, with the largest differences in CV_Glx/tCr_ (*P* < 0.01). The reproducibility of metabolites quantified with lower precision (i.e., higher CRLBs) was similarly reduced by maximum 3% when using the param MM basis set in comparison to the full MM (Supporting Information Figure S2). The mean CVs were <12% for Glu/tCr, <15% for GSH/tCr, and <20% for Gln/tCr and Tau/tCr among all brain regions for full MM basis set. The reproducibility of Gln/tCr was significantly improved when using full MM basis set in comparison to no MM (*P* < 0.001). Generally, the CVs were comparably low between the brain regions in both MRSI acquisitions, and only CVs_Glx/tCr_ were slightly higher in the subcortical WM (by ~3%, *P* < 0.001) and in the WM regions (by ~2%, *P* < 0.01) than in the GM regions, likely due to the lower signal intensity of Glx in the WM. Similarly, CV_mIns/tCr_ was increased by ~2% in the subcortical WM (*P* < 0.01) compared to the other 4 regions.

**Figure 5:**
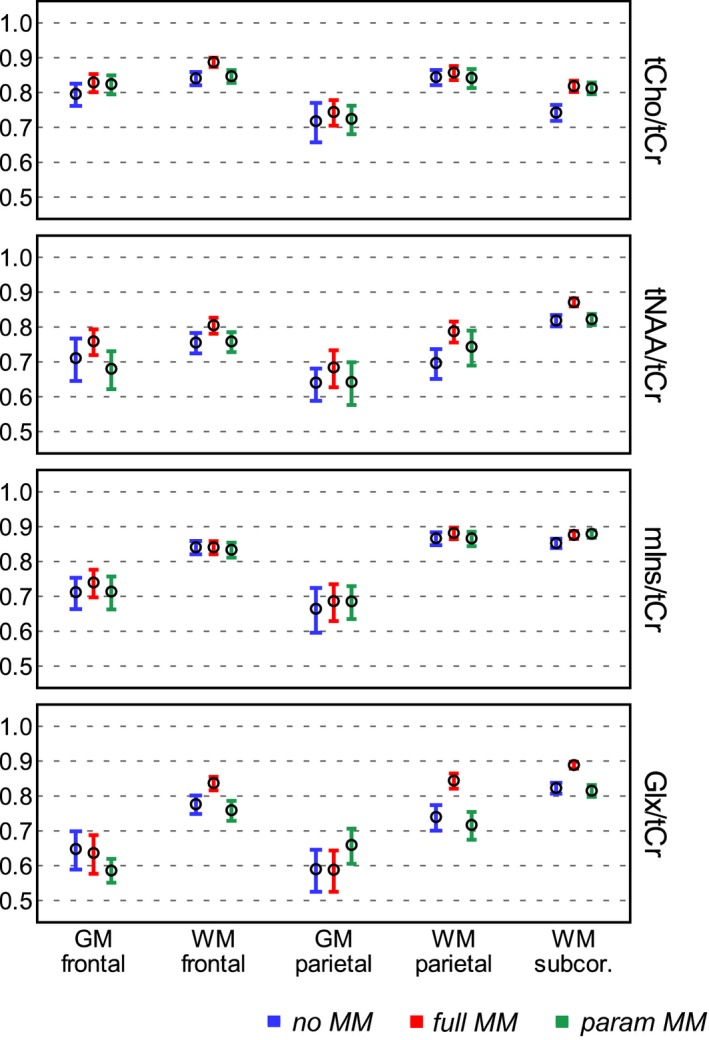
ICC of metabolite ratios obtained by FID‐MRSI and quantified by 3 different basis sets: no MM,with no macromolecular information; full MM, with a single measured macromolecular spectrum; and param MM, with 9 individual macromolecular components with soft constraints. The circles and the error bars represent the mean and 95% confidence interval of the total number of analyzed voxels. ICC, intraclass correlation coefficients

ICC values confirmed a very good agreement between the measurements. Generally, the highest ICC values were in the WM regions when using the full MM basis set (mean ± SD in WM regions, ICC_tCho/tCr_ = 0.85 ± 0.07, mean ICC_tNAA/tCr_ = 0.80 ± 0.09, ICC_mIns/tCr_ = 0.85 ± 0.07, ICC_Glx/tCr_ = 0.83 ± 0.08). Overall, the obtained ICCs indicate moderate (0.5 < ICC < 0.75) to good (0.75 < ICC < 0.9) reliability between the test and retest measurements in both MRSI acquisitions. The ICC values were comparably high between the basis sets (mean ± SD, ICC_noMM_ = 0.76 ± 0.08, ICC_fullMM_ = 0.79 ± 0.09, ICC_paramMM_ = 0.74 ± 0.09). The incorporation of MM information into the fitting process either via a single spectrum or over 9 parameterized MM peaks had no negative effect on the reliability of MRSI; in fact, all ICC_fullMM_ ≥ ICC_noMM_ and only ICC_paramMM_ of Glx/tCr (of both MRSI acquisitions) in the frontal GM or the frontal WM were significantly decreased (*P* < 0.01) compared to ICC_noMM_ (Figure 5 and Supporting Information Figure S3).

## 4 DISCUSSION

This study compares the effect of using 3 different macromolecular models on the test–retest reproducibility of brain metabolite quantification for 7T FID‐MRSI. The comparison was performed between basis sets with no MM information, with a single measured MM spectrum, and with 9 individual MM peaks included in the fitting prior knowledge.

Based on obtained CVs and ICCs, we provide evidence that FID‐MRSI can map the spatial distribution of 4 neurometabolite ratios with high precision, particularly when using the full MM model (CV ≤ 8%, ICC ≥ 0.76) in only ~5 min. The inclusion of individual MM components into the quantification and using adequate soft constraints resulted in almost the same reproducibility but no systematic absolute error compared to the quantification using full MM, and it also offered the added benefit of full flexibility in case of (un‐)expected local changes in the MM profile. We found no strong correlation between individual MM components and overlapping metabolites that would indicate a severe bias due to unintentional overfitting. This indicates that the full MM model may be superior for healthy volunteer studies or those in which no major changes in the MM profile are expected, but the difference in reproducibility is small enough to make the param MM fitting model the preferred choice when the MM composition is expected to change, for example, in pathologies. In such cases, fitting MM resonances individually should help maintain the accurate quantification of metabolites and even macromolecules themselves at the expense of only slightly reduced reproducibility. This could provide additional diagnostic information.

We achieved very good spectral quality and quantification precision, which further support the validity of the FID‐MRSI method. The small increase (~1%) in CRLB when param MM basis set was used is probably caused by increased number of degrees of freedom of the model. Several metabolite ratio levels were fairly decreased when incorporating MM prior knowledge into the fitting model. These differences were in good agreement with previously published results in which the tNAA, Glu, mIns, tCr, and tCho signals were decreased by approximately 25%, 15%, 13%, 5%, and 8% in the GM and by 16%, 12%, 12%, 7%, and 11% in the WM when using single measured MM spectrum in the basis set.^17^ Thus, the largest changes can be found in the GM of tNAA/tCr. The strong correlations we observed between some of the MM resonances can explain a possible physiologically meaningful relation between these resonances, or these dependencies could be partially attributable to the soft constraints applied. Moreover, MM resonances at 1.22, 1.43, and 1.70 ppm can be affected by the lipid signal removal.

The reproducibility of brain spectroscopic methods have been investigated extensively among different field strengths and acquisition techniques.^10^, ^33^, ^34^, ^35^, ^36^, ^37^, ^38^, ^39^, ^40^, ^41^, ^42^, ^43^, ^44^ Previous short TE 7T single‐voxel MRS reproducibility studies have differed in their approaches to handle the MM contributions by using either the automatic MM calculation of LCModel,^35^ the group‐averaged measured MM spectrum (here, full MM) as part of the basis set,^36^ the inversion‐based suppression of the MM signal,^10^ or no MM correction at all.^34^ These studies were conducted with STEAM or semi‐LASER using a 3 to 27 cm^3^ voxel volume and TEs ranging from 14 to 72 ms positioned in the anterior cingulate, posterior cingulate, prefrontal, or occipital cortex. The CVs for the same metabolites as reported in our work were below 5% when using the measured MM spectrum and below 10% when using LCModel's MM calculation or suppression of macromolecules.

To our best knowledge, studies concerning long TE MRS(I) reproducibility were conducted at lower field strengths only. According to Birch et al.^14^ and Inglese et al.,^45^ the use of intermediate/long TE outperformed short TE in terms of reproducibility, except of Glu (or Glx) and mIns, which had better reproducibility when using short(er) TE. The CVs obtained in their works at 3T using PRESS localization (with long or intermediate TE) were comparable to or worse than our CVs from FID‐MRSI at 7T.

Birch et al. also compared in their work the influence of experimental and simulated MM models on the MRSI reproducibility at 3T^14^ and concluded that the use of experimental MM basis sets resulted in a slightly better performance than the use of simulated macromolecules. The CV < 6% for tNAA, tCr, and tCho (for TE = 80ms) and the CV < 16% for Glx and mIns (for TE = 35ms) were comparable to or worse than our results from 7T. In contrast, Schaller et al. showed that using a mathematical approximation model for the MM contribution was sufficient at 3T; however, the reproducibility of metabolite quantification through CVs was not assessed.^12^

Another parameter, ICC, is a commonly used reliability index in test–retest analysis. ICC depends not just on the measurement errors of the method but also on the true heterogeneity in the population. Ideally, the variability in measurements is due to genuine differences between the subjects; then, ICC = 1. However, when ICC = 0, the observed variability is only a measurement error. The slightly lower ICC values of tNAA/tCr compared to those of tCho/tCr or mIns/tCr can be caused by imperfect lipid decontamination, which mostly affects the NAA peak. MRS reproducibility studies performed at 7T lack information about ICC and primarily concentrate on CV. Compared to the spin‐echo MRSI sequence at 3T,^33^ we achieved better reliability in the WM (the mean ICC_WM_ > 0.8 vs. ICC_WM_ > 0.55) and comparable reliability in the GM (mean ICC_GM_ > 0.7). Thus, our results show that neither extracranial lipids nor MM contributions significantly reduced reproducibility compared to other reports, although both are enhanced in FID‐MRSI, especially when highly accelerated by parallel imaging. A further reduction of lipid artifacts via dedicated lipid removal hardware^46^ or spatial‐spectral encoding^47^ is expected to further improve the reproducibility.

The limitations of our study include the absence of absolute metabolite concentrations, which would allow a more direct comparison of our results with previous works. Absolute quantification requires information about water density and relaxation times of the metabolites. Our sequence benefits from negligible T_2_‐weighting; however, it is sensitive to incorrect assumptions about T_1_ relaxation times due to the relatively short TR of 600 ms. Therefore, we decided to report metabolite levels with respect to tCr, which is a commonly used internal reference peak. Due to the lack of ground truth, only the precision and not the accuracy of the method could be assessed. The LCModel's internal MM quantification was not included in our comparison because of the FID acquisition and the resulting phase problems. Fitting range was reduced for no MM analysis; otherwise, having unfitted peaks in residuum would cause wrong CRLB estimation. The reproducibility was evaluated in 2 axial MRSI slices at different levels, both avoiding the deep brain structures, which are usually strongly affected by spatial B0 inhomogeneity. Nevertheless, we achieved very good reproducibility in the frontal lobe, which is similarly considered challenging for B_0_ shimming. Due to restricted scanner availability, we could not maintain the same interval between the measurements for all the subjects. Some of the repeated measurements were performed on a different day, which could have resulted in biological differences between the datasets, but these should be negligible for young healthy volunteers.^48^

## 5 CONCLUSION

FID‐MRSI with in vivo measured macromolecular contribution included in the fitting prior knowledge provides highly reproducible quantification for common neurometabolites at 7T in only ~5 min. The use of the whole measured MM spectrum provided the highest reproducibility in young healthy volunteers. Parameterization of the MM spectrum yielded only slightly lower reproducibility compared to a single MM component; however, parameterization may be beneficial when the MM profile is expected to be altered due to pathological changes. This makes FID‐MRSI a feasible clinical research tool to target brain biochemistry as well as for applications beyond investigations of the common brain metabolites.

## Supporting information


**FIGURE S1** Coefficient of variations (CV) of the second MRSI slice acquired at the upper level of the brain
**FIGURE S2** Coefficient of variations (CV) of the metabolites quantified with lower quantification precision
**FIGURE S3** ICC of the second MRSI slice acquired at the upper level of the brain
**TABLE S1** Metabolite ratios quantified by LCModel using three different basis sets
**TEXT S1** Control files for LCModel quantification
