# Supplementary material for: Effects of different macromolecular models on reproducibility of FID‐MRSI at 7T
Source: Magn Reson Med. 2019 Aug 8;83(1):12–21. doi: 10.1002/mrm.27922 (PMC6851974; doi:10.1002/mrm.27922)
Supplement: Supplementary file 1 — FIGURE S1 Coefficient of variations (CV) of the second MRSI slice acquired at the upper level of the brain FIGURE S2 Coefficient of variations (CV) of the metabolites quantified with lower quantification precision FIGURE S3 ICC of the second MRSI slice acquired at the upper level of the brain TABLE S1 Metabolite ratios quantified by LCModel using three different basis sets TEXT S1 Control files for LCModel quantification [file MRM-83-12-s001.docx]

**Supporting Information Text S1:** Control files for LCModel quantification

1. Control file for LCModel quantification using *full MM* basis set, which includes single measured macromolecular spectrum.

WSMET = ''DSS'''

WSPPM = 0.0

N1HMET = 9

SUBBAS = F

NEACH = 99

DKNTMN = 0.15

PPMST = 4.2

PPMEND = 0.2

DEGZER = 0

SDDEGZ = 20

DEGPPM = 0

SDDEGP = 20

NSIMUL = 0

CPU_cores = 9

LTABLE = 7

LCSV = 0

LCOORD = 9

1. Control file for LCModel quantification using *param MM* basis set, which includes nine individual macromolecular resonances with soft constraints defined with the LCModel parameter “CHRATO”.

WSMET = ''DSS'''

WSPPM = 0.0

N1HMET = 9

SUBBAS = T

NEACH = 99

DKNTMN = 0.15

PPMST = 4.2

PPMEND = 0.2

DEGZER = 0

SDDEGZ = 20

DEGPPM = 0

SDDEGP = 20

NRATIO = 10

CHRATO(1) = 'MM_1_2/MM_0_9 = 0.12 +- 0.10'

CHRATO(2) = 'MM_1_4/MM_0_9 = 0.235 +- 0.15'

CHRATO(3) = ''MM_1_6/MM_0_9 = 0.24 +- 0.3'

CHRATO(4) = ''MM_2_0/MM_0_9 = 0.74 +- 0.25'

CHRATO(5) = ''MM_2_2/MM_0_9 = 0.6 +- 0.3'

CHRATO(6) = ''MM_2_9/MM_0_9 = 0.30 +- 0.2'

CHRATO(7) = ''MM_3_2/MM_0_9 = 0.32 +- 0.2'

CHRATO(8) = ''MM_3_7/MM_0_9 = 1.01 +- 0.3'

CHRATO(9) = ''MM_2_9/MM_3_2 = 0.99 +- 0.1'

CHRATO(10) = ''MM_2_2/MM_2_0 = 0.81 +- 0.1'

NSIMUL = 0

CPU_cores = 9

LTABLE = 7

LCSV = 0

LCOORD = 9

1. Control file for LCModel quantification using *no MM* basis set, which includes neither measured nor simulated macromolecular resonances. Hence, only the spline baseline was used to compensate for the macromolecular signals.

WSMET = ''DSS'''

WSPPM = 0.0

N1HMET = 9

SUBBAS = F

NEACH = 99

DKNTMN = 0.15

PPMST = 4.2

PPMEND = 1.8

DEGZER = 0

SDDEGZ = 20

DEGPPM = 0

SDDEGP = 20

NSIMUL = 0

CPU_cores = 9

LTABLE = 7

LCSV = 0

LCOORD = 9

**Supporting Information Table S1:** Metabolite ratio levels obtained by LCModel quantification using three different basis sets.

| **tNAA/tCr** | **GM** | **WM** |
| --- | --- | --- |
| *no MM* | 1.76±0.20 | 1.92±0.25 |
| *full MM* | 1.53±0.18^*^ | 1.76±0.22^*^ |
| *param MM* | 1.56±0.22^*^ | 1.80±0.26^*^ |
|  |  |  |
| **tCho/tCr** | **GM** | **WM** |
| *no MM* | 0.30±0.04 | 0.39±0.06 |
| *full MM* | 0.28±0.04 | 0.38±0.07 |
| *param MM* | 0.29±0.05 | 0.38±0.07 |
|  |  |  |
| **mIns/tCr** | **GM** | **WM** |
| *no MM* | 0.80±0.10 | 0.92±0.19 |
| *full MM* | 0.75±0.10 | 0.86±0.21^*^ |
| *param MM* | 0.73±0.11^*^ | 0.84±0.18^*^ |
|  |  |  |
| **Glx/tCr** | **GM** | **WM** |
| *no MM* | 1.58±0.23 | 1.19±0.28 |
| *full MM* | 1.52±0.22 | 1.13±0.28^*^ |
| *param MM* | 1.47±0.26^*†^ | 1.11±0.31^*^ |

*Note*: Mean ± standard deviations are provided. ^*^ indicates significant to *no MM* and ^†^ significant to *full MM.*

Abbreviations: GM, grey matter; WM, white matter

**
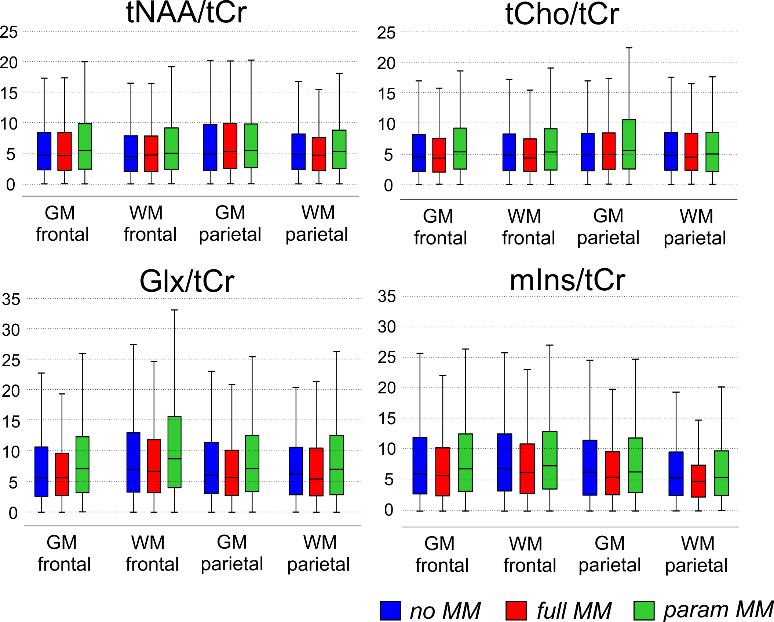
**

**Supporting Information Figure S1:** Coefficient of variations (CV) of the second MRSI slice acquired at the upper level of the brain.


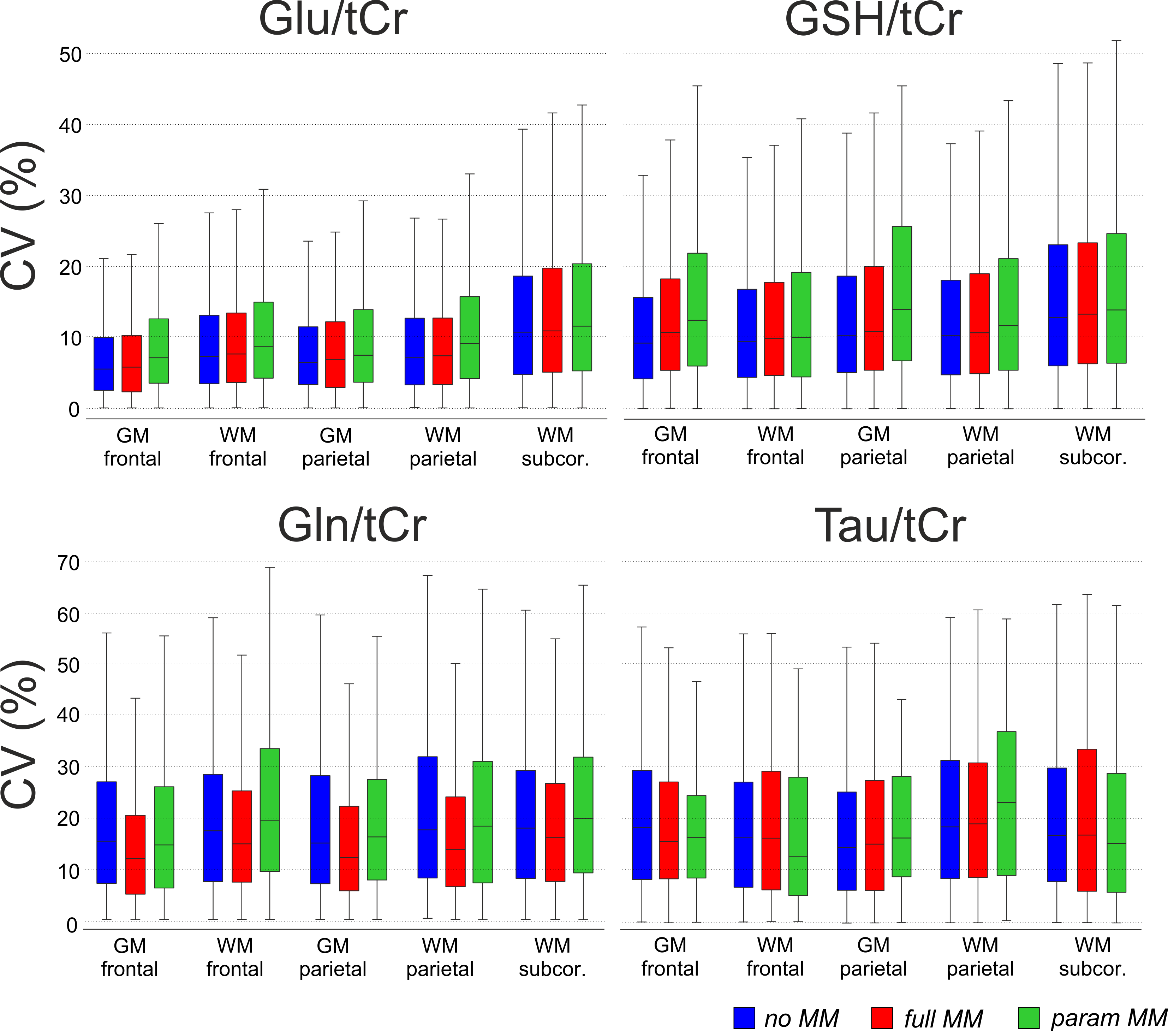


**Supporting Information Figure S2:** Coefficient of variations (CV) of the metabolites quantified with lower quantification precision.


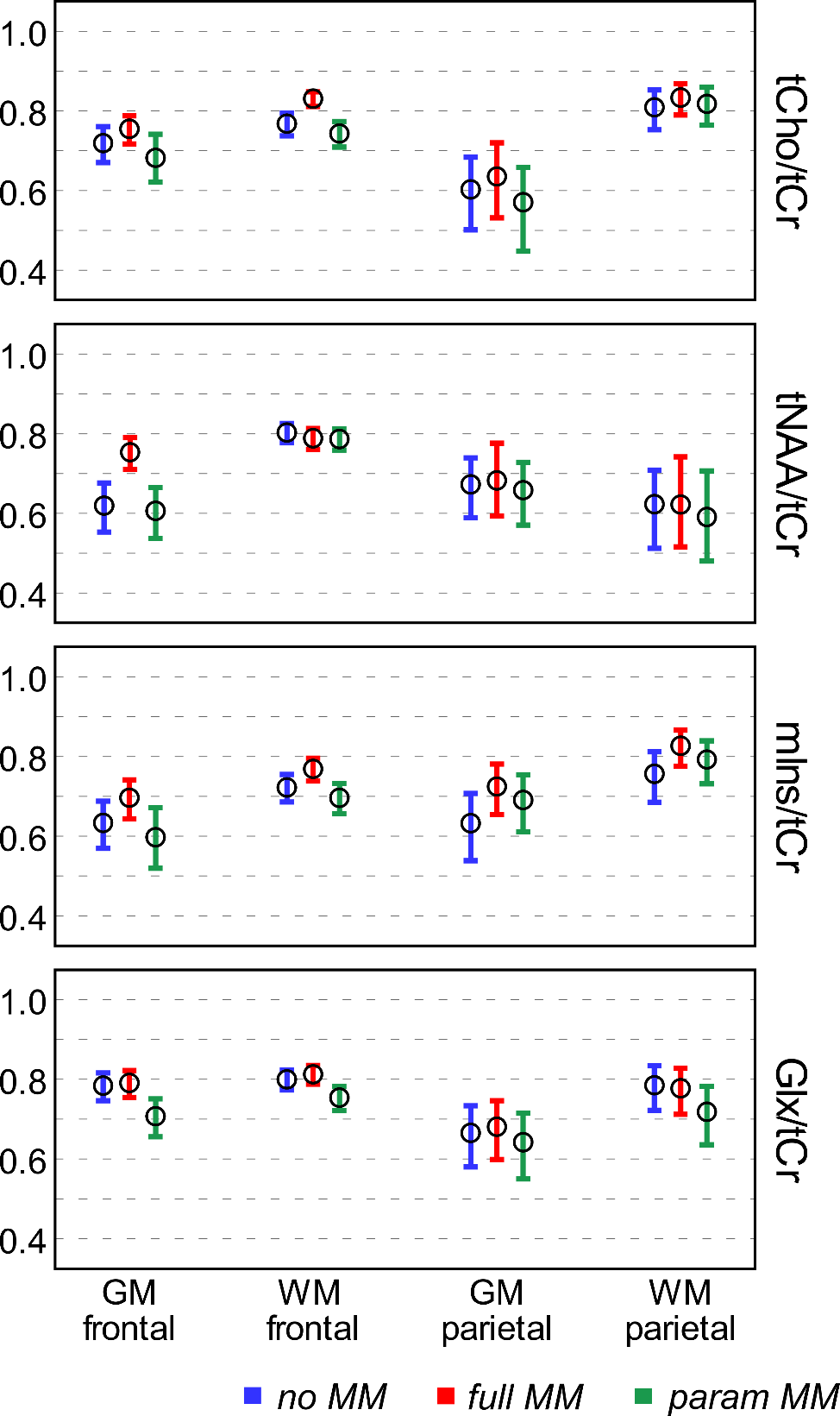


**Supporting Information Figure S3:** ICC of the second MRSI slice acquired at the upper level of the brain.
